# Antiamoebic Properties of Ceftriaxone and Zinc-Oxide–Cyclodextrin-Conjugated Ceftriaxone

**DOI:** 10.3390/antibiotics11121721

**Published:** 2022-11-30

**Authors:** Zinb Makhlouf, Noor Akbar, Naveed Ahmed Khan, Muhammad Raza Shah, Ahmad M. Alharbi, Hasan Alfahemi, Ruqaiyyah Siddiqui

**Affiliations:** 1 College of Arts and Sciences, American University of Sharjah, University City, Sharjah 26666, United Arab Emirates; 2 Department of Clinical Sciences, College of Medicine, University of Sharjah, University City, Sharjah 27272, United Arab Emirates; 3 Department of Medical Biology, Faculty of Medicine, Istinye University, Istanbul 34010, Turkey; 4 H.E.J. Research Institute of Chemistry, International Centre for Chemical and Biological Sciences, University of Karachi, Karachi 75270, Pakistan; 5 Department of Clinical Laboratory Sciences, College of Applied Medical Sciences, Taif University, Taif 21944, Saudi Arabia; 6 Department of Medical Microbiology, Faculty of Medicine, Al-Baha University, Al-Baha 65799, Saudi Arabia

**Keywords:** *Acanthamoeba castellanii*, zinc oxide, nanotechnology, drug discovery, CNS infections, free-living amoebae

## Abstract

*Acanthamoeba castellanii* is a ubiquitous free-living amoeba capable of instigating keratitis and granulomatous amoebic encephalitis in humans. Treatment remains limited and inconsistent. Accordingly, there is a pressing need for novel compounds. Nanotechnology has been gaining attention for enhancing drug delivery and reducing toxicity. Previous work has shown that various antibiotic classes displayed antiamoebic activity. Herein, we employed two antibiotics: ampicillin and ceftriaxone, conjugated with the nanocarrier zinc oxide and β-cyclodextrin, and tested them against *A. castellanii* via amoebicidal, amoebistatic, encystment, excystment, cytopathogenicity, and cytotoxicity assays at a concentration of 100 μg/mL. Notably, zinc oxide β-cyclodextrin ceftriaxone significantly inhibited *A. castellanii* growth and cytopathogenicity. Additionally, both zinc oxide β-cyclodextrin ceftriaxone and ceftriaxone markedly inhibited *A. castellanii* encystment. Furthermore, all the tested compounds displayed negligible cytotoxicity. However, minimal anti-excystment or amoebicidal effects were observed for the compounds. Accordingly, this novel nanoconjugation should be employed in further studies in hope of discovering novel anti-*Acanthamoeba* compounds.

## 1. Introduction

*Acanthamoeba castellanii* (*A. castellanii*) is a ubiquitous free-living amoeba isolated from soil, dust, treated and untreated water, air-conditioning units, contact lenses, ophthalmological solutions, dialysis units, surgical material, fecal material, human throats mucosa, and even air [1,2,3,4]. This amphizoic microorganism is a reported etiological agent of a frequently misdiagnosed sight-threating corneal infection, *Acanthamoeba* keratitis, resulting from corneal abrasion due to exposure to heavily contaminated liquid [1,2]. Importantly, the number of *Acanthamoeba* keratitis cases has been on the rise despite advances in antimicrobial chemotherapy and support care [5]. This is likely due to the increasing numbers of contact lens users and/or global warming associated with outdoor water-related activities/poor compliance in the use of contact lenses. After gaining entry hematogenously, through the lower respiratory tract, skins ulcers, or through the olfactory epithelium, *Acanthamoeba* can also bring about a fatal brain infection mainly in immunocompromised patients: granulomatous amoebic encephalitis [2,4]. Notably, *Acanthamoeba* can also engender skin nodules and abscesses [6]. While anti-*Acanthamoebic* drugs are available such as biguanides, namely polyhexamethylene biguanide and chlorhexidines, these therapeutic measures display inconsistent efficacy and high toxicity [1]. Furthermore, under adverse conditions, *Acanthamoeba* transforms from a vegetative trophozoite to a resistant double-walled cyst, which enables it to withstand severe conditions including disinfectant and antimicrobial agent administration [1]. Hence, this amoeba is highly resistant to antiamoebic drugs [6]. In this light, there is a pressing need for novel antiamoebic drugs with minimal side effects. 

With their small size and large surface to volume ratio, nanoparticles have recently proven effective against various microorganisms such as fungi, viruses, bacteria, and parasites [7]. Moreover, metallic oxide nanoparticles such as zinc oxide display reactive oxygen species (ROS) induction, surface functionalization with peptides, a marked safety profile, and efficacy against *A. castellanii* [7,8,9]. Through interrupting the protein synthesis cycle at different stages, several antibiotic classes have been reported to display tropical and cytocidal activities against *Acanthamoeba* [9]. Ceftriaxone (CFT) is a third-generation cephalosporin with notable penetration in most body fluids including cerebrospinal fluid and is used as a first-line treatment for various infections such as meningitis [10]. Similarly, ampicillin (AMPI) is a β-lactam antibiotic belonging to the penicillin class employed to treat cardiovascular, gastrointestinal, and urinary infections [11]. Cyclodextrin is a cyclic oligosaccharide linked by 1,4-glycosidic bonds, and with its hydrophobic cavity, it increases the water solubility of various small molecules [12]. In this light, we employed each of the antibiotics conjugated with the nanocarrier zinc-oxide–cyclodextrin in this study. Notably, cyclodextrins exhibit different physicochemical properties with β-cyclodextrin existing as nanoparticles in solution. β-cyclodextrin having 07 glucopyranose units was used in the current study. β-cyclodextrin is one of the most widely used biocompatible and biodegradable systems. It is also widely used as an excipient in formulation [13]. β-cyclodextrin is known to generate metal nanoparticles with a narrow size distribution as compared to α and γ cyclodextrin. The narrow size distribution of metal nanoparticles is known to play an important role for enhanced biological activities. Additionally, the cavity size of β-cyclodextrin lies in the range of 7 angstrom, which is the best fit for molecules having six members; the ceftriaxone, which was used as the host in this study, contained two six-member cyclic rings, and the β-cyclodextrin appeared to be a good host. Similarly, β-cyclodextrin also tends to form micelles in solution, which is important for penetration into the cell membranes of parasites. For simplicity, β-cyclodextrin is referred to as CD throughout the manuscript. 

Moreover, amoebicidal, amoebastatic, amoebastatic MIC50, encystment, excystment, cytopathogenicity, and cytotoxicity assays were carried out to determine the efficacy of ZnO-CD-CFT and ZnO-CD-AMPI against *A*. *castellanii.* Accordingly, these nanocarriers show promise in the quest for novel contact lens disinfectants against *A*. *castellanii.*

## 2. Methodology

### 2.1. Nanocarriers and Nanoconjugates Employed

As can be seen in (Table 1), the drugs were prepared as described by our group in a previous study [14,15]. Briefly, the nanoparticles were prepared through the direct precipitation method starting from zinc acetate dihydrate and NaOH. In brief, quercetin, naringin, and β-cyclodextrin were acquired from Sigma-Aldrich, whereas the amphotericin, ampicillin, and ceftriaxone were obtained from Merck; the solutions were prepared using deionized water. Zinc oxide nanoparticles were produced using the direct precipitation method, utilizing zinc acetate dihydrate and NaOH as precursors. Nanoparticles and drug conjugates were characterized to determine their stability using UV-visible spectrophotometry, dynamic light scattering, Fourier transform infrared spectroscopy, and atomic force microscopy [14]

### 2.2. Cultures of A. castellanii

*A. castellanii* from the American Type Culture Collection 50492, genotype T4, were cultured, as detailed previously [6,16,17]. In short, the amoebae were cultured and maintained in proteose peptone 0.75%, glucose 1.5%, and yeast extract 0.75% (PYG) medium axenically at a temperature of 30 °C until confluency was observed. All experiments were conducted at room temperature.

### 2.3. Cultures of Henrietta Lacks (HeLa) Cervical Cancer Cells

Henrietta Lacks (HeLa) cells from the American Type Culture Collection (ATCC CCL-2) were grown at 37 °C and 5% CO_2_ in Roswell Park Memorial Institute (RPMI)-1640 enriched with 1% minimal essential media nonessential amino acid (MEM NEAA), 10% fetal bovine serum, 1% l-glutamine, and 1% penicillin–streptomycin [18,19,20]. Where a monolayer was needed, HeLa cells were transformed to well plates, which engendered mature monolayers in 48 h [20].

### 2.4. Amoebicidal Assays

Amoebicidal assays were carried out as outlined previously [16,17,21]. Firstly, *A. castellanii* (5 × 10^5^ amoeba/mL per well) were treated with each of the compounds (Table 1) at a dosage of 100 μg/mL, using RPMI as a medium at 37 °C. After an incubation period of 24 h, a Trypan blue assay was employed to enumerate viable amoebae. As a negative and positive control, amoebae in RPMI and amoebae in 0.25% SDS were used, respectively. 

### 2.5. Amoebistatic Assays

Amoebistatic assays were carried out as detailed in previous work [22,23]. Briefly, *A. castellanii* (5 × 10^5^ amoeba/mL per well) were incubated with each compound (Table 1) for 24 h using PYG as a medium at 37 °C. Amoebae alone in PYG were used as the negative control. For the positive control, amoebae incubated in sodium dodecyl sulfate (SDS) were used. Viable amoebae were enumerated using hemocytometer counting and compared with each of the controls. Finally, using concentrations of 50, 75, and 100 µM, the MIC_50_ values for the amoebastatic effects were determined [24].

### 2.6. Encystation Assays

To investigate the effects of the employed compounds on *A. castellanii* encystation, encystation assays were carried out as outlined previously [24,25,26]. In short, a count of 1 × 10^6^ amoebae was incubated with a concentration of 100 μg/mL of each compound (Table 1) in filtered 16% glucose, with amoebae alone in glucose and amoebae with SDS in glucose serving as negative and positive controls, respectively at 37 °C. Cyst formation was checked until a 48-h incubation period was completed. Afterwards, 0.1% SDS was added to each well for 20 min to ensure that only cysts were being counted. Finally, the cysts were enumerated with a hemocytometer. 

### 2.7. Excystation Assays

To prepare cysts, *A. castellanii* trophozoites were placed grown on non-nutrient agar for 2 weeks at 30 °C [27,28]. Afterwards, the cysts were harvested by scarping the non-nutrient agar in autoclaved distilled water, after which they were centrifuged, and the resulting cyst pellet was suspended in PYG. As described in detail previously, 1 × 10^5^ cysts were incubated with a concentration of 100 μg/mL of each compound (Table 1) in PYG at 30 °C for 24–48 h [27]. The positive control was amoeba with SDS in PYG. The negative control, which was amoebae in PYG alone, was monitored to ensure complete excystation. Finally, the emerging trophozoites were enumerated using a hemocytometer and compared with both controls. 

### 2.8. Cytopathogenicity Assays

To investigate the effects of each compound on *A. castellanii*-mediated host cell cytopathogenicity, cytopathogenicity assays were carried out [28,29]. Briefly, a 2-h amoebicidal assay was carried out with each of the compounds (Table 1) at a concentration of 100 μg/mL, and instead of counting the viable trophozoites, the adherent cells were detached from the 24-well plates, centrifuged, and added to a monolayer of HeLa cells to carry out a lactate dehydrogenase (LDH) assay. Moreover, after an incubation of 24 h, the released LDH was quantified and compared with the negative control of HeLa cells alone and the positive control of amoebae and Triton X-100. LDH is an intracellular enzyme, which is only detected if released from damaged cells. Notably, amoebae-mediated host cells’ damage is dependent on the amoeba strain as well as the inoculum size. In our experiments, amoebae-mediated damage was observed optimally following 24 h of incubation. 

### 2.9. Cytotoxicity Assays

To investigate the toxicity of each compound (Table 1) towards human cells, cytotoxicity assays were executed as formerly described [16,30,31]. Briefly, HeLa cells were grown until adequate confluency was observed and were then incubated with a concentration of 100 μg/mL of each compound. An LDH kit was used to quantify the resulting cell damage, and a positive control of HeLa cells with Triton X-100 and a negative control of HeLa cells alone were used to facilitate comparison. 

### 2.10. Statistical Analysis

To determine statistical significance for differences between duplicates, a two-sample t-test with two-tailed distribution was carried out [31,32,33]. Moreover, the means of two individual experiments repeated under similar conditions were compared to determine the *p* values using Microsoft Excel.

## 3. Results

### 3.1. ZnO-CD-CFT, CFT, ZnO-CD-Control, ZnO-CD-AMPI, and AMPI Displayed No Significant Amoebicidal Activity in 24 h of Incubation at a Concentration of 100 μg/mL

**Figure 1:**
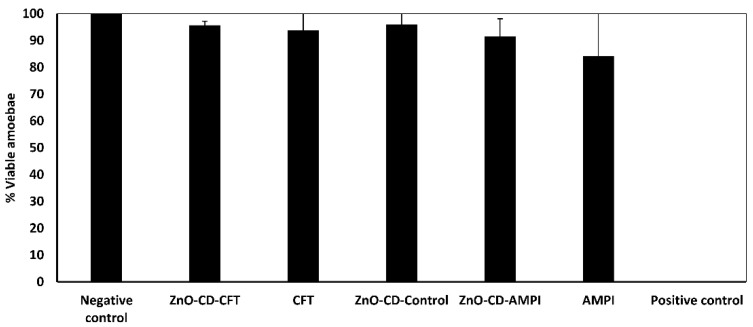
The amoebicidal activity of the compounds was investigated. After a 24 h incubation period, all compounds showed no amoebicidal effects of significance at the concentration of 100 μg/mL. The results are presented as the mean ± standard error.

To investigate whether the employed compounds possessed any amoebicidal activity against *A. castellanii*, amoebicidal assays were executed. The positive control was considered 0%, and the negative control was considered 100% for visualization ease. None of the compounds displayed amoebicidal activity at the employed concentration of 100 μg/mL (Figure 1). Furthermore, after an incubation period of 24 h, the ZnO-CD-CFT, CFT, ZnO-CD-Control, and ZnO-CD-AMPI reduced amoebic viability by merely 10%. Similarly, the AMPI reduced amoebic viability by no more than 20%. Hence, none of the compounds displayed amoebicidal activity against *A. castellanii* at the concentration of 100 μg/mL in 24 h in comparison to the controls. 

### 3.2. ZnO-CD-CFT Markedly Inhibited the Growth of A. castellanii at a Concentration of 100 μg/mL in 24 h of Incubation

**Figure 2:**
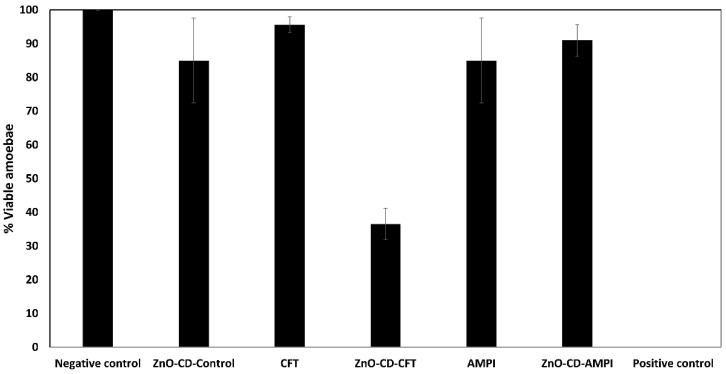
The amoebistatic effects of the compounds were tested. ZnO-CD-CFT markedly inhibited the growth of *A. castellanii* at a concentration of 100 μg/mL in 24 h of incubation. The other compounds showed no amoebistatic effects of significance. The results are presented as the mean ± standard error. Note that ZnO-CD-CFT but not CFT alone showed significant inhibition (*p* < 0.05, using a two-sample *t* test, two tailed distribution).

**Figure 3:**
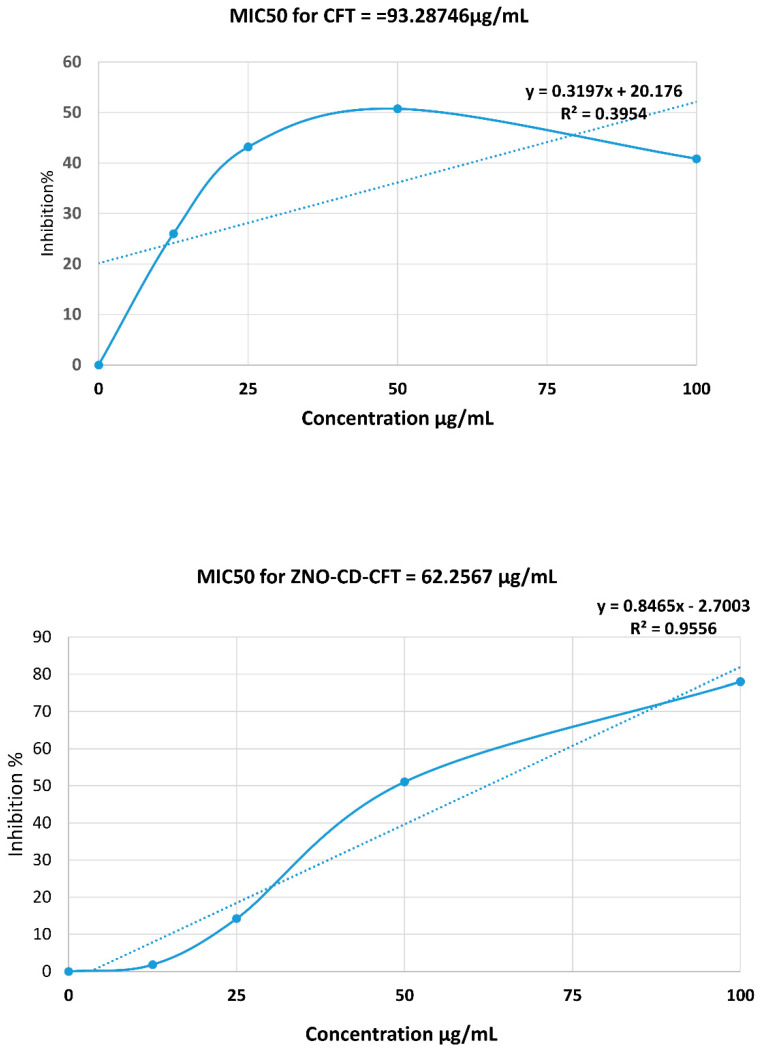
The MIC_50_ values of the CFT and ZnO-CD-CFT for amoebistatic activity were determined using the following concentrations: 50, 75, and 100 µM. The MIC_50_ values for the CFT and ZnO-CD-CFT were 101.9 μg/mL and 74.64 μg/mL, respectively. The results are presented as the mean ± standard error.

To determine whether the employed compounds had any amoebistatic effects against *A. castellanii*, amoebistatic assays were carried out. As depicted (Figure 2), the ZnO-CD-CFT displayed marked effects on *A. castellanii* growth in comparison to the negative control, which was 100% amoebic viability, and the positive control, which was 0% viability. Moreover, after incubation for 24 h in PYG, the ZnO-CD-CFT reduced amoebic viability by approximately 65% with statistical significance. Conversely, the ZnO-CD-Control, CFT, AMPI, and ZnO-CD-AMPI did not possess any amoebistatic effects of significance. Furthermore, the ZnO-CD-Control reduced amoebic viability by almost 20%, the CFT by less than 10%, the AMPI by almost 20%, and the ZnO-CD-AMPI by around 10%. Thus, the ZnO-CD-CFT displayed marked amoebistatic effects against *A. castellanii* in 24 h, at a concentration of 100 μg/mL. The MIC_50_ values were also determined and were 93 μg/mL for CFT and 62 μg/mL for ZnO-CD-CFT as depicted (Figure 3). 

### 3.3. ZnO-CD-CFT and CFT Markedly Inhibited the Encystment of A. castellanii at a Concentration of 100 μg/mL in 48 h of Incubation

**Figure 4:**
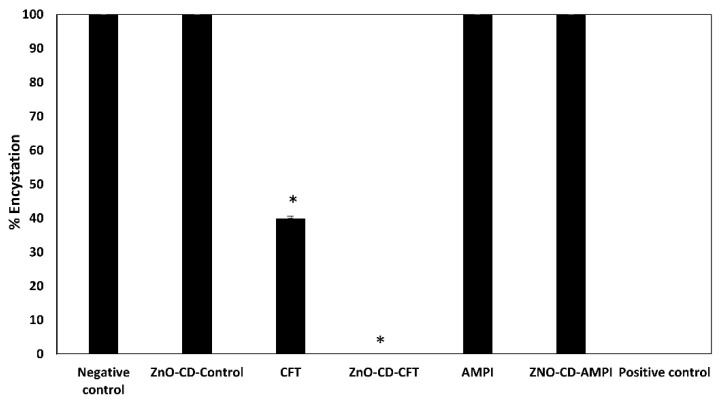
To determine whether the utilized compounds inhibited *A. castellanii* encystation, encystment assays were carried out. The results revealed that the CFT and ZnO-CD-CFT markedly inhibited encystment at a concentration of 100 μg/mL in 48 h incubation. The data are presented as the mean ± standard error (* is *p* < 0.05).

Encystment assays were executed to pinpoint whether the utilized compounds inhibited *A. castellanii* encystment. The negative and positive controls were 100% and 0% amoebic viability, respectively. As depicted (Figure 4), the ZnO-CD-CFT displayed marked effects against *A. castellanii* encystment. Furthermore, the ZnO-CD-CFT reduced encystment by approximately 100% with statistical significance. Similarly, the CFT showed statistically significant effects, reducing *A. castellanii* encystment by around 60%. On the other hand, the ZnO-CD-Control, AMPI, and ZnO-CD-AMPI showed no effects on encystment. Moreover, they reduced the encystment by less than 10%. Accordingly, the ZnO-CD-CFT and CFT displayed notable effects against *A. castellanii* encystment at a concentration of 100 μg/mL after 48 h of incubation. 

### 3.4. ZnO-CD-CFT and CFT Displayed no Statistically Significant Effects against A. castellanii Excystment

**Figure 5:**
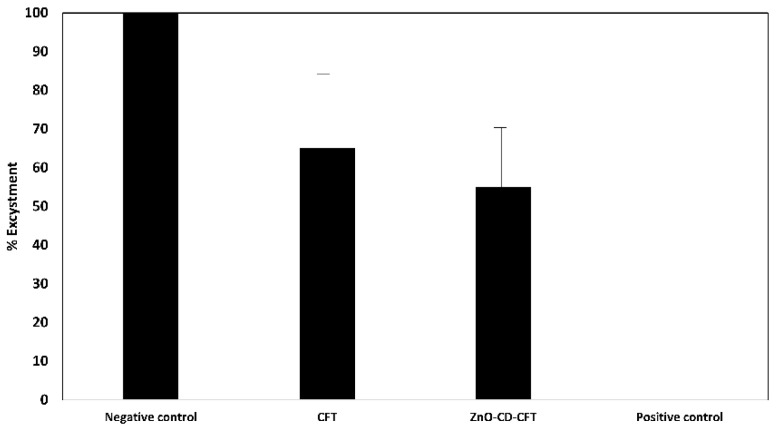
After observing the marked effects of the ZnO-CD-CFT and CFT, excystment assays were performed to investigate the effects of the CFT and ZnO-CD-CFT on *A. castellanii* excystation. At the concentration of 100 μg/mL, ZnO-CD-CFT and CFT showed no statistically significant effects on excystation. The results are presented as the mean ± standard error.

In the light of the observed efficacy of the ZnO-CD-CFT and CFT in previous assays, excystment assays with these two compounds were carried out. As elucidated (Figure 5), the CFT and ZnO-CD-CFT reduced the number of emerging trophozoites; yet, that reduction was not statistically significant in comparison to the negative control, which was 100% viability, and the positive control, which was 0% viability. The CFT reduced excystment by approximately 40%, and the ZnO-CD-CFT reduced excystment by 46% without statistical significance. Accordingly, the ZnO-CD-CFT and CFT did not display statistically significant effects against *A. castellanii* at a concentration of 100 μg/mL in 24–48 h of incubation (Figure 5). 

### 3.5. ZnO-CD-CFT Significantly Reduced A. castellanii Cytopathogenicity

**Figure 6:**
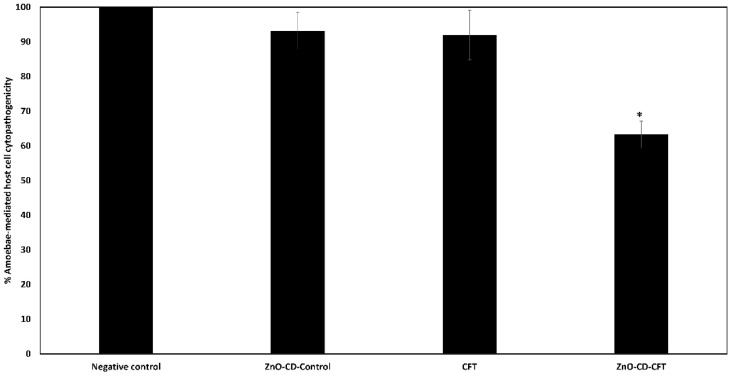
Cytopathogenicity assays were executed to investigate the effects of the ZnO-CD-CFT, CFT, and ZnO-CD-Control on *A. castellanii* mediated cytopathogenicity. Notably, the ZnO-CD-CFT reduced *A. castellanii* cytopathogenicity with significance. The results are presented as the mean ± standard error (* is *p* < 0.05).

To test whether these drugs retained their efficacy in conditions similar to that of a real infection, cytopathogenicity assays were carried out. Notably, the ZnO-CD-CFT reduced *A. castellanii* cytopathogenicity with statistical significance when compared with the negative control, which was *A. castellanii* and HeLa cells in RPMI. As can be seen (Figure 6), the ZnO-CD-CFT reduced *A. castellanii* cytopathogenicity by approximately 38% with statistical significance. Conversely, the ZnO-CD-Control and CFT merely reduced it by 7% and 9%, respectively. Accordingly, the ZnO-CD-CFT was able to significantly reduce *A. castellanii* mediated host pathogenicity.

### 3.6. All Tested Compounds Displayed Low Cytotoxicity

**Figure 7:**
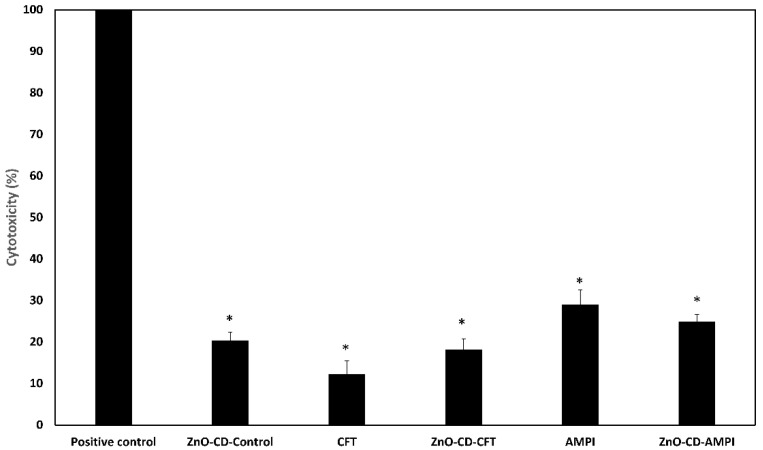
Cytotoxicity assays were performed to determine the toxicity of these compounds towards human cells. All the employed compounds displayed minimal cytotoxicity. The results presented as the mean ± standard error (* is *p* < 0.05).

Cytotoxicity assays were executed to determine the safety of the employed compounds for human use. Notably, all the tested compounds displayed negligible cytotoxicity. As can be seen (Figure 7), all the employed compounds had a cytotoxicity below 30%. Moreover, the ZnO-CD-Control, CFT, ZnO-CD-CFT, AMPI, and ZnO-CD-AMPI had a cytotoxicity of approximately 20%, 12%, 18%, 29%, and 25%, respectively. Based on the International Organization for Standardization, (ISO) 10993-5, if cell viability is within 60% to 80%, then limited cytotoxic activity is present [34,35]. Hence, all the employed compounds exhibited low cytotoxicity in comparison to the positive control, which was 100% cytotoxicity.

## 4. Discussion

*A. castellanii* is a reported etiological agent behind the serious human infections, *Acanthamoeba* keratitis and granulomatous amoebic encephalitis, which are increasing in prevalence [16,36]. Notably, these infections present clinical challenges despite advances in antimicrobial therapy [37]. This is plausible since the presence of one *A. castellanii* cyst within the host tissue is all that is needed to drive reactivation [36]. Currently utilized antiamoebic compounds include diamidines, biguanides, and antifungal azole derivatives, which are aggressive and highly toxic [38]. Accordingly, the quest for novel anti-*Acanthamoebic* compounds is of paramount importance. 

Inorganic nanoparticles including metal nanoparticles have been incorporated in various medical applications due to their affordable production, versatility, low toxicity, and robustness [39]. When it comes to amoebae, several nanoparticles bearing antibiotics, namely silver- and gold-based nanocarriers have proved efficacious against amoebae [31,39,40,41] Other studies employed tannic acid-modified silver, azole, dysprosium-based, and cobalt phosphate nanoparticles and reported marked anti-*Acanthamoebic* activity [42,43,44,45]. Due to its potency against bacteria, the nanocarrier ZnO has been implemented in various biomedical applications including water treatment [46]. Several antibiotics classes were observed to exhibit cytocidal activities against *Acanthamoeba* [9]. Yet, clinically it is still unclear whether antibiotics are effective as adjunct therapeutic measures against *A. castellanii* [47,48]. Accordingly, in light of the previously reported efficacy of CFT and AMPI in conjugation with ZnO and the cyclic oligosaccharide with hydrophilicity enhancing effects: CD against bacteria, we tested this nano-conjugation against *A. castellanii* [12,14]. 

Our results revealed that while no amoebicidal activity was observed by any of the compounds (Table 1), ZnO-CD-CFT markedly inhibited the cytopathogenicity and growth of *A. castellanii* at a concentration of 100 μg/mL after 24 h of incubation. In terms of *A. castellanii* encystment, both CFT and ZnO-CD-CFT notably reduced encystation within 48 h at a concentration of 100 μg/mL, yet both compounds were unable to inhibit *A. castellanii* excystation. Notably, all the employed compounds displayed minimal cytotoxicity at this concentration. To the best of our knowledge, this is the first time that this specific nanocarrier formulation has been tested against *A. castellanii*. Yet, our findings are consistent with previous work reporting ZnO negligible toxicity and its efficacy against bacterial pathogens [14]. While further research is needed to pinpoint the mechanism behind the efficacy enhanced efficacy of CFT when conjugated with ZnO-CD against *A. castellanii*, we speculate several points. Firstly, the mechanisms behind ZnO nanoparticles are not elucidated but are speculated to be the disruption of the cell membrane, release of metallic ions, and generation of ROS [49]. Secondly, CFT is a cephalosporin with marked penetration in most body fluids including cerebrospinal fluid, whose mode of action is through inhibiting bacterial cell wall synthesis [10,14]. Hence, given CFT’s ability to inhibit encystment as shown from our results, CFT may have inhibited the formation of the double-walled hardy cyst. Regardless, the action of the CFT was magnified and even in some assays only efficacious when conjugated with ZnO-CD. Hence, future mechanistic studies are required to disprove, prove, or introduce alternative theories behind this efficacy. 

This work demonstrated the efficacy of the cephalosporin CFT when conjugated with the metal nanocarrier ZnO-CD against *A. castellanii.* Future work could address the limitations of this project, namely employing variations of incubation periods and dosages, using in vivo models, and using confocal microscopy to visualize the mechanism behind the antiamoebic activity of this formulation. 

## Figures and Tables

**Table 1:** This table outlines the nanocarriers and drugs used in this study with their abbreviations.

Abbreviation	Full Name
ZnO-CD-Control	Zinc Oxide β-cyclodextrin
CFT	Ceftriaxone
ZnO-CD-CFT	Zinc Oxide β-cyclodextrin ceftriaxone
ZnO-CD-AMPI	Zinc Oxide Ampicillin
AMPI	Ampicillin
Negative control	Amoebae alone in medium
Positive control	Amoebae with 0.25% sodium dodecyl sulfate (SDS) in medium

## Data Availability

The data presented in this study are available on request from the corresponding author.
